# Contrasting seasonal and spatial distribution of native and invasive *Codium* seaweed revealed by targeting species‐specific eDNA

**DOI:** 10.1002/ece3.5379

**Published:** 2019-07-10

**Authors:** Teja Petra Muha, Roberta Skukan, Yaisel J. Borrell, José M. Rico, Carlos Garcia de Leaniz, Eva Garcia‐Vazquez, Sofia Consuegra

**Affiliations:** ^1^ Swansea University Swansea UK; ^2^ Neoalgae Asturias Spain; ^3^ Department of Functional Biology University of Oviedo Oviedo Spain; ^4^ Department of Biology of Organisms and Systems (BOS) University of Oviedo Oviedo Spain

**Keywords:** barcoding, *Codium* spp., environmental DNA, invasive species, rbcL, real‐time PCR, tufA

## Abstract

**Aim:**

*Codium fragile*, an invasive seaweed, has spread widely during the last century, impacting on local seaweed communities through competition and disturbance. Early detection of *C. fragile* can help on its control and management. Environmental DNA (eDNA) has proved successful for early detection of aquatic invasive species but its potential use for seaweed remains understudied. We used a species‐specific eDNA qPCR approach to investigate the spatial distribution, abundance, and coexistence of the invasive *C. fragile* and three native *Codium* species (*Codium vermilara*, *Codium tomentosum*, and *Codium decorticatum*) in the Cantabrian Sea.

**Location:**

Bay of Biscay, Northern Atlantic Coast of the Iberian Peninsula; two ports, a beach and a rocky cliff.

**Methods:**

We designed species‐specific primers in barcoding regions targeting short fragments of the rbcL gene for the invasive *Codium* species, and the elongation factor Tu (tufA) gene for the native species, to assess their spatial and seasonal distributions using quantitative real‐time PCR in samples collected during summer, autumn, and winter.

**Results:**

We found seasonal differences in the presence of the invasive *Codium fragile* and two of the native *Codium* species, but did not detect *C. decorticatum* at any point. Species distribution patterns produced with qPCR targeting species‐specific eDNA coincided with the known distribution based on previous conventional sampling, with a seasonal alternance of *C. fragile* and *C. vermilara*, and a marked dominance of invasive *C. fragile* in ports, which are known hotspots for invasive species.

**Main conclusions:**

Our results demonstrate the utility of using eDNA for early detection and monitoring of invasive seaweed. Native and invasive *Codium *spp. displayed significant seasonal and spatial differentiation that needs to be taken into account in risk management. Regular monitoring of ports and adjacent areas using eDNA should help to assess the potential expansion of invasive *Codium* and the need for management interventions to avoid the displacement of native seaweed.

## 1 INTRODUCTION

The invasive seaweed *Codium fragile* has been regarded as one of the four most damaging seaweed invaders (Provan, Murphy, & Maggs, 2005), displacing local seaweed communities by its opportunistic physiological adaptations (Scheibling & Gagnon, 2006) and changing the structure of faunal assemblages (Drouin, McKindsey, & Johnson, 2011). *C. fragile* is accidentally introduced to new localities as a fouling organism on ships’ hulls (Carlton & Scanlon, 1985; Drouin & McKindsey, 2007) and can easily spread by currents before becoming established on the coast (Carlton & Scanlon, 1985). Ports are known hotspots for invasive species (Drake & Lodge, 2004) and can potentially host more dense populations of invasive *C. fragile* in comparison with natural locations without artificial structures, which facilitate their growth (Bulleri & Airoldi, 2005). The invasive green seaweed *Codium fragile *ssp.* fragile* (Suringar) Hariot (hereafter *C. fragile*) has become established on the intertidal shores of the Cantabrian Sea (northwestern Spain; García, Olabarria, Arrontes, Álvarez, & Viejo, 2018), coexisting with native *C. tomentosum* Stackhouse, *C. vermilara* (Ollivi) Delle Chiaje, and *C. decorticatum* (Woodward) Howe (Juanes, Guinda, Puente, & Revilla, 2008; Martínez‐Gil, Gallardo, Díaz, & Bárbara, 2007; Skukan et al., 2017), with *C. fragile* being the only present subspecies identified in the area (Rojo et al., 2014). Recruitment of *C. fragile* in the Bay of Biscay relies on newcomers rather than on established populations’ vegetative regeneration (García et al., 2018), implying that higher densities of invasive seaweed are likely found in ports.

Cryptic invasion of morphologically similar invasive and native species (Provan, Booth, Todd, Beatty, & Maggs, 2008) has been identified as the most plausible cause for the competition between *C. fragile* and the native *Codium *spp. (García et al., 2018). Due to the wide physiological adaptations of *C. fragile* and its preference for higher temperatures during the reproductive season (Hanisak, 1979), new potential niches for its settlement are proliferating under current climatic conditions (Zanolla & Andreakis, 2016). Spatio‐temporal information of native and invasive *Codium *spp. is crucial for evaluating whether patterns of competitive displacement or coexistence take place in Cantabrian Sea, where rising sea‐surface temperatures have favored the spread of warm‐water nonindigenous species over the past three decades (Díez, Muguerza, Santolaria, Ganzedo, & Gorostiaga, 2012).

Until now, knowledge on the spatial and seasonal distribution of seaweed has relied on traditional sampling methods (García et al., 2018), based on physical specimen collection and taxonomic identification, either based on morphological traits or molecular sequencing. These methods are typically limited by the feasibility of collecting specimens depending on the tides and weather conditions (Rojo et al., 2014), as well as by the multiple reproductive patterns of the different species (Schmidt & Scheibling, 2005). In addition, the taxonomic identification of different *Codium *spp. based on phenotypic traits is particularly challenging (Zanolla & Andreakis, 2016), often requiring molecular identification. Therefore, a more rapid and accurate detection tool is needed to monitor and/or control the distribution of invasive seaweed, which is less weather and tide dependent and incorporates the advantages of molecular identification.

Early detection allows rapid response to eradicate or limit the spread of aquatic invasive species (AIS; Jerde, Mahon, Chadderton, & Lodge, 2011). Detection of species using environmental DNA (eDNA) is noninvasive and can identify species presence by isolating genetic material from their surrounding environment (Thomsen & Willerslev, 2015) and is increasingly being used for detection of AIS (Dejean et al., 2012; Piaggio et al., 2014; Takahara, Minamoto, & Doi, 2013). Species‐specific eDNA assessment by PCR or qPCR can be used for presence/absence detection as well as for relative abundance estimates, providing comparable estimates to traditional sampling techniques (Dejean et al., 2012; Doi et al., 2015; Takahara et al., 2013). eDNA has proved useful for the detection of aquatic invertebrates (Deiner, Fronhofer, Mächler, Walser, & Altermatt, 2016; Mächler, Deiner, Steinmann, & Altermatt, 2014) and vertebrates (Piaggio et al., 2014; Sigsgaard et al., 2016; Takahara et al., 2013), but the information on the aquatic plants and algae is still limited. Only a few studies have addressed the detectability of aquatic plants or algae with eDNA (Fujiwara, Matsuhashi, Doi, Yamamoto, & Minamoto, 2016; Keller, Hilderbrand, Shank, & Potapova, 2017; Scriver, Marinich, Wilson, & Freeland, 2015; Zimmermann, Glöckner, Jahn, Enke, & Gemeinholzer, 2015), due to the limited availability of reference databases (Cristescu, 2014) and the lineage‐specific barcodes (Zanolla & Andreakis, 2016). To be useful for detecting seaweed, eDNA barcodes need to be specific (Verbruggen et al., 2010) and have a suitable resolution across multiple regions (Zanolla & Andreakis, 2016) within the suspected introduced range of targeted taxa (Geller, Darling, & Carlton, 2010). Given the increase in invasion rates worldwide (Ruiz, Carlton, Grosholz, & Hines, 1997), the use of eDNA has the potential to revolutionize the detection of cryptic invasive seaweed, which has been rarely assessed until now.

Early detection of spatial and temporal changes in the distribution of *Codium *spp. is essential for assessing the potential displacement of native seaweeds in the Bay of Biscay. We evaluated the extent of seasonal and spatial overlap between native and non‐native intertidal Codium seaweed. We also investigated whether invasive Codium was more frequent in ports than in natural coastal locations, in order to identify potential areas for targeted containment management.

## 2 METHODS

### 2.1 Study sites

**Figure 1:**
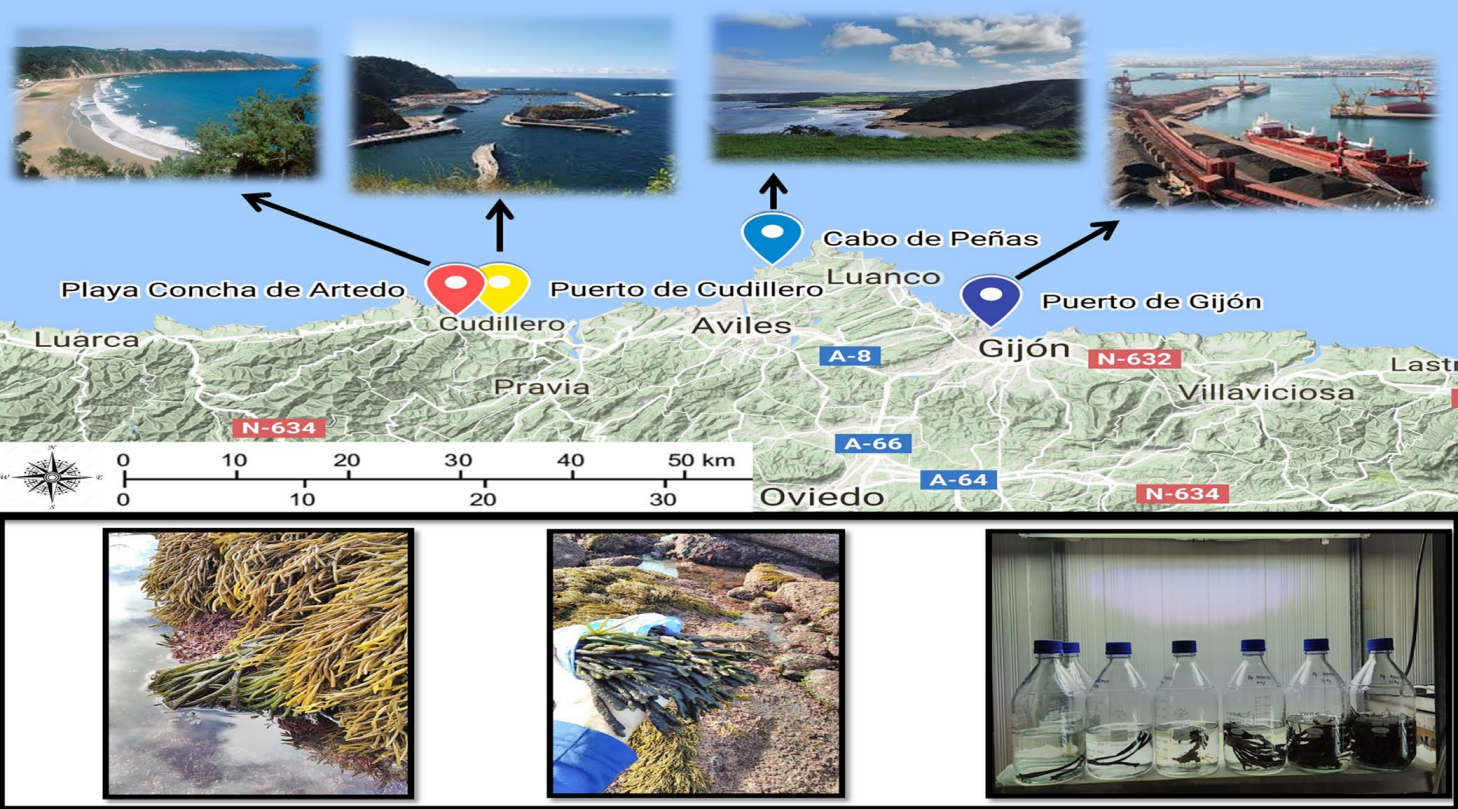
(a) DNA sampling locations from west to east side: Concha de Artedo, small port of Cudillero, rocky intertidal platform Cabo de Peñas, and international port of Gijón; (b) collection of *C. tomentosum* specimens and layout of the eDNA mesocosm experiment. The selected images of natural localities and ex situ experiment belong to authors, and the images of ports were collected from the Google marked with permission for reuse and modifications

Water samples were collected in July, October, and December 2017 at four different stations in Asturias (N. Spain) including a sandy beach with few rock formations, Concha de Artedo (latitude 43°34′01.7″N, longitude 6°11′29.5″W), the small port of Cudillero (latitude 43°34′02.1″N, longitude 6°09′04.1″W), the rocky cliff Cabo de Peñas (latitude 43°37′31.3″N, longitude 5°53′48.5″W), and the large international port of Gijón (latitude 43°33′18.3″N, longitude 5°41′25.9″W) (Figure 1). The sampling covered 40.26 km of coast. Samples for Cabo de Peñas were not available in July. Average water temperatures in all three sampling months (July, October, and December) were 21.9, 20.6, and 15.8°C in Gijón and 21.5, 20.2, and 15.6°C in Cudillero. We recorded seawater temperature in situ at Concha de Artedo and Cabo de Peñas using two Hobo Temperature Loggers (Onset Computer Corporation) permanently fixed to the substratum at an average height of 1 m below mean sea level, with measured 22.2*°*C maximum summer seawater temperature (SST) at Concha de Artedo and 21.7*°*C at Cabo de Peñas, and 12.4 and 12.0*°*C minimum winter temperatures at both stations, respectively. There was a difference of 0.4–0.5*°*C, increasing toward east based on average monthly SST.

### 2.2 Ex situ experiment

An ex situ experiment was designed focusing on *C. tomentosum* to validate primer efficiency based on eDNA copy number with species density. The experimental setup consisted of six presterilized glass bottles with 1 L of marine water to which different densities (5, 10, 20, 40, 80, 160 g) of *C. tomentosum* were added and one control containing only seawater. Specimens were collected at Cabo de Peñas in October 2017 and brought in a cooling bag back to the laboratory, where they were identified morphologically following Provan et al. (2008), gently dried, and weighted before being added to the experimental 1‐L water bottles (Figure 1b). The increase of *C. tomentosum* biomass was based on doubling the previous weight to test for a correlation between eDNA quantity assessed by qPCR (Ct values) and species biomass. The marine water for the experiment was collected at a location with no known presence of *C. tomentosum*. Water temperature was kept between 16 and 17.5*°*C. *C. tomentosum* specimens were kept in bottles for 36 hr and removed afterward. The water from the bottles was filtered using the same eDNA filtering procedure as described below for each bottle separately. The negative filtration control using sterile nuclease‐free water was filtered first, followed by filtration of marine water only, and then the rest of the bottles containing *C. tomentosum* in order of concentration, starting by the lowest. The DNA was extracted using the same protocol as for the collected eDNA water samples from field described below, including an additional negative extraction control, with extractions being stored at −20*°*C.

### 2.3 Environmental DNA collection, filtration, and extraction

Three replicates of water samples (1 L of each) were collected with sterile bottles approximately 30 cm under the surface at all sampling sites at consistent sampling points for each of the three sampling periods (Figure 1a). All four sites were sampled either on the same day or in two consecutive days. Nitrile gloves were used while collecting the water. A cooling bag was used for the transportation to the laboratory where filtration took place immediately after returning from the field. Filtering in the laboratory took place in a dedicated eDNA room, where steps were taken to avoid eDNA contamination following Goldberg et al. (2016). A filter funnel was used for vacuum filtering in combination with sterile Supor1‐200 Membrane Disc Filter (Pall Corporation) with 0.2 μm pore size. Water flow was 70 kPA. For each of the sampling replicates, a maximum of two filters were used and stored together in a separate tube from other replicates at −20°C until the next day when DNA extraction was processed. A negative control sample was filtered using sterile nuclease‐free water between filtration samples from different sampling locations. DNA was extracted on the following day of filtrations using the PowerWater^®^ DNA Isolation Kit Sample (Qiagen GmbH) following the manufacturer's recommendations with a modified last step of 50 µl for DNA elution. DNA extraction took place in a pressurized fume hood dedicated solely to eDNA handling. Sampling triplicates were extracted individually, including all five negative filtration controls with an additional negative control extraction sample for each of the sampling seasonal periods. DNA extractions were stored at −20*°*C before further processing.

### 2.4 Primer design and validation

We developed species‐specific primers in barcoding regions (rbcL and tufA genes) for the seasonal and spatial assessment of the invasive species *C. fragile* in coexistence with native *Codium *spp. We targeted 364 bp of the rbcL gene chloroplast subunit for the invasive *C. fragile* based on reference nucleotide sequences from GenBank, as this gene has previously been used for species identification (Verbruggen et al., 2007). For the three native species *C. tomentosum*, *C. vermilara*, and *C. decorticatum,* 211‐, 180‐, and 249‐bp short fragments of plastid elongation factor Tu (tufA) gene were targeted to design species‐specific markers (Table 1). The plastid tufA and rbcL markers are some of the most widely applied markers to taxonomically separate the green algae group (Saunders & Kucera, 2010; Škaloud, Kynčlová, Benada, Kofroňová, & Škaloudová, 2012). To test the species specificity of the primers, they were firstly tested in silico using Primer‐BLAST (Ye et al., 2012) and afterward used to amplify and cross‐amplify tissue samples of the individual species before being used on eDNA samples for PCR and qPCR. First, primers were optimized for PCR, then for use in qPCR, where detection limits were determined. Cross‐species amplifications were tested on each individual species amplifying it with all four primer pairs. *C. decorticatum* primers could not be tested on this species as no specimens were found along the Asturian coast at the time of the research. Extraction mixtures contained several specimens of each individual species to account for intraspecies variability. Tissues were extracted using GeneMATRIX Plant and Fungi Purification Kit (GeneMATRIX Purification Kit, Roboklon GmbH). A 100‐fold dilution of an initial 1 ng/µl of each tissue was used for cross‐amplifications in order to mimic eDNA detection levels in the environment. All specimens of *C. fragile* collected in the Bay of Biscay region were identified based on sequencing as the invasive subspecies *C. fragile *ssp. *fragile* (Rojo et al., 2014), confirming the primer specificity for the subspecies. Oligo Analyser 3.1 tool (Integrated DNA Technologies) was used for primer check on hairpins and primer dimers. To estimate the detection sensitivity of each specific primer pair, 10‐fold serial dilutions, starting from 1 ng/µl down to 1:10,000,000, were used and limits of detection were defined by qPCR amplification using dilution triplicates, for all three species individually. The last detectable melt peak at each species‐specific melt temperature was accounted as detection limit and reported as corresponded dilution level. Additionally, the same 10‐fold dilution was applied for defining the qPCR standard curve.

**Table 1:** Species‐specific PCR primers used for amplification of targeted chloroplast rbcL and tufA region, with reported sequence, amplicon size (including primers), annealing temperature, qPCR detection limit based on 10‐fold dilution series, and specific PCR and qPCR running conditions

Target species	Primer	Sequence (5′–3′)	Amplicon size (bp)	Annealing PCR (T *°*C)	qPCR detection limit (ng/µl)	Melt peak (*°*C)	Annealing qPCR (T *°*C)
*C. fragile ssp. fragile*	C. fragRBCL F	ACATTCTTGCAGCTTTTCGT	364	58	1 × 10^−4^	82	65
	C. fragRBCL R	TTCATCCCATGAGGTGGTC					
*C. tomentosum*	C. tomCDS F	AACCAGCTTCTATTTTACCCCA	211	56	1 × 10^−4^	79.5	65
	C. tomCDS R	TCCATTTGAATACGATCTCCCG					
*C. vermilara*	C. verCDS F	CGCCATTTTCAAGCACAGGTA	180	57	1 × 10^−6^	78	65
	C. verCDS R	AATTCGATCTCCCGGCATTAC					
*C. decorticatum*	C. decorCDS F	TACAGGAAGGGGTACGGTTG	249	57	/	/	65
	C. decorCDS R	TGTCGATGAGGCATAATAGAAGC					

Abbreviation: bp: base pair.

### 2.5 PCR amplification

PCR and qPCR were optimized to avoid cross‐species amplification for each specific primer pair. PCR conditions were as follows: 7 min at 95*°*C, followed by 10 touchdown cycles of 95*°*C for 30 s, 68–58*°*C for 30 s, 72*°*C for 30 s, with additional 15 cycles of 95*°*C for 30 s, 58*°*C for 30 s, 72*°*C for 30 s, and a final extension step at the 72*°*C for 5 min. For *C. vermilara*, *C. tomentosum*, *C. fragile*, and *C. decorticatum*, the annealing temperature was 57, 56, 58, and 57*°*C, respectively (Table 1, Figure S1). The amplification reaction for the PCR included 1× Colorless GoTaq^®^Buffer, 2.5 mM MgCl_2_, 1 mM dNTPs, 50 pmol of each primer, 0.5 U of DNA Taq Polymerase (Promega), 0.2 μg/μl BSA, and 3 μl of eDNA with nuclease‐free water added up to total volume of 20 μl. The same PCR conditions were used for both, tissue and eDNA samples, with the only difference in the number of annealing cycles, 25 for tissue and 40 cycles for eDNA. For positive controls, tissues were diluted down to 0.1 ng/µl including tested 10× and 100× fold dilutions to define primer efficiency on eDNA dilution level. PCR products were visualized on 2% agarose gel with added 2 μl of SimplySafe™. All PCR products were directly sequenced using Sanger sequencing at Macrogen Europe (Spain). Sequences were confirmed for each specific species by BLAST. Negative filtration and extraction samples were amplified using the same procedures.

For the quantification of each individual species from the eDNA samples, real‐time PCR (qPCR) was performed using SYBR Green technology (Bio‐Rad). The reaction mixture contained 1× SsoAdvanced™ Universal SYBR^®^ Green Supermix, 25 pmol of forward and reverse primer, and 3 µl of extracted DNA with additional nuclease‐free water to the final volume of 20 µl with all amplifications run out on a 96‐well reaction plate (Bio‐Rad) including triplicates of negative control PCR where nuclease‐free water was added instead of the template, as well as triplicates of positive controls added to each run. All species‐specific amplifications were run on separate plates. All eDNA samples were run in triplicate. Additional cross‐species assessment was evaluated through qPCR with all four primers tested on all three different tissues. The qPCR conditions were as follows: 10 min at 95*°*C, followed by 10 s at 95*°*C and 30 s at 65*°*C, in 35 cycles total for all four species. A melting curve was included at the end of qPCR run within a range of 60 to 95*°*C. Data were analyzed with Bio‐Rad CFX Manager (Bio‐Rad).

### 2.6 eDNA absolute quantification

In order to compare the seasonal and spatial distribution between the three species, absolute quantification based on differences in eDNA copies was performed, calibrated by each specific qPCR run efficiency. Absolute quantification determines the input copy number by correlating PCR signal to a standard curve (Schmittgen & Livak, 2008). Each individual species’ copy number estimate was determined by the exact copy concentration of the target gene correlated to Ct values according to the standard curve (Lee, Kim, Shin, & Hwang, 2006) as used previously in eDNA studies (Dougherty et al., 2016; Renshaw, Olds, Jerde, McVeigh, & Lodge, 2014), by firstly calculating the number of copies per each individual species‐specific targeted DNA length, using Avogadro's number (6.022 × 10^23^ molecules/mole) and a general assumption that the average weight of a base pair (bp) is 650 Daltons as calculated by Whelan, Russell, and Whelan (2003), following:

$$\text{DNA}\;\left(\text{copy}\;\text{number}\right)=\left(6.02\times 10^{23}\left(\text{copy/mol}\right)*\text{DNA}\;\text{concentration}\;\left(\text{ng/}\mu l\right)\right)/\left(\text{DNA}\;\text{length}\;\left(\text{bp}\right)\times 650\;\left(\text{g/mol/bp}\right)\right).$$

The DNA copy number was used for calculation of the initial concentration given for the standard curve. Each standard curve was performed by a linear regression of the plotted standards. The slope of each standard curve determines qPCR efficiency (*E*), calculated by the following equation Lee et al. (2006):

$$E=10^{-1/\text{slope}}-1.$$

From the copy number of each standard, we quantified each sample by relating Ct values to the standard curve (Yu, Lee, Kim, & Hwang, 2005). Each specific sample quantification was performed as in Gallup (2011):

$$\text{Absolute}\;\text{copy}\;\text{number}\left(\text{eDNA}\;\text{copies}\right)=E^{\left(\text{Standard}\;\text{curve}\;\text{intercept}-\text{Sample}\;\text{average}\;Ct\;\text{value}\right)}.$$

All eDNA copy numbers were estimated per microlitre of filtered water (eDNA copies/µl).

### 2.7 Statistical analysis

We modeled presence/absence data and species density in relation to season, sampling site, and artificial/natural locations applying linear models. The two ports (Gijon and Cudillero) and two natural locations (Concha de Artedo and Cabo de Peñas) were grouped together by artificial/natural categories to test for differences between origins of sampling localities. For presence/absence data, we employed a binary logistic regression within two models, firstly assessing interactions between species, location, and sampling season, and secondly the interactions between species, sampling season, and type of location (natural/artificial). At least two positive detections (out of three sampling replicates) were considered sufficient as evidence of presence. To model abundance, we used a linear model with a Gaussian error distribution to investigate variation in eDNA copies/µl as function of species, location, and sampling season in first model and species, sampling season, and natural/artificial location in the second model, including their interactions. For the post hoc analysis, the “lsmeans” package was used (Lenth, 2016) based on Tukey contrasts. The qPCR triplicates of each of the three sampling replicates were averaged before statistical analysis. In the case that one of the sampling triplicates did not amplify and the other two did, the amplification of sampling triplicates was repeated for confirmation, with at least two sampling replicates used for further statistical analysis. For estimation of efficiency in species‐specific models, as well for comparison of abundance among species, the eDNA copies/µl were used. For the ex situ experiment, a Pearson correlation coefficient was carried out (Benesty, Chen, Huang, & Cohen, 2009), between eDNA copy numbers (based on Ct values) depending on *C. tomentosum* actual biomass (g/l). All statistical analyses were done with the program R, version 3.3.2, with “dplyr” and “ggplot2” package used for data representation.

## 3 RESULTS

In total, 132 eDNA qPCR triplicates, 11 filtering, and three extraction negative controls were used for qPCR quantification. In seven of the samples, not all three sampling replicates produced species‐specific positive confirmations, five targeting *C. tomentosum* and two targeting *C. fragile*; thus, sampling duplicates were used for further analysis. Triplicates of 21 eDNA samples, two filtrations, and one extraction negative controls from ex situ experiment were additionally processed for individual assessment based on correlation between *C. tomentosum* eDNA copies/µl and species density (g/l). There was no in silico possible cross‐contamination for any of the species (native or invasive), tested with the Primer‐BLAST tool on NCBI page (Johnson et al., 2008). No cross‐amplification was produced either in PCR or in qPCR for any of the three species tested with all four primer sets, using dilution series of the three target species *C. tomentosum*, *C. fragile*, and *C. vermilara*.

Negative controls produced no amplification in any cases. Both controls from the ex situ experiment, the marine water, and nuclease‐free water did not amplify during PCR and qPCR tested with all four primer pairs. All positive controls confirmed the target species by accurate alignment to sequences from target species, using BLAST and BioEdit (Hall, 1999). In total, four individual forward and reverse sequences for all three primer sets on *C. vermilara*, *C. fragile*, and *C. tomentosum* were used for measures of primers’ efficiencies as positive controls on species’ tissue extractions. In total, 81 eDNA samples were sequenced, 30 for *C. tomentosum*, 29 for *C. vermilara*, and 22 for *C. fragile*, confirmed by 98%–100% similarity rate in BLAST, with nine unique sequences added to the GenBank under the nucleotide accession numbers (MK503248‐MK503252, MK503325‐MK503328, MK507407‐MK507412). *C. decorticatum* did not amplify in any of the qPCR triplicates of 132 eDNA samples and was not considered for further analysis.

For qPCR cross‐amplification, no melt peaks were observed using cross‐referenced primers on species‐specific target samples, confirming the specificity of the primers. Melt peaks of the three target species *C. fragile*, *C. tomentosum*, and *C. vermilara* were at 82, 79.5, and 78*°*C, respectively (Table 1). For the invasive *C. fragile*, the qPCR quality run resulted in *R* ^2^ = 0.97 based on the standard curve approach, with an efficiency of 99% and a slope of −3.345. For the native *C. tomentosum*, the qPCR run resulted in *R* ^2^ = 0.991 with an efficiency of 99.9% and a slope of −3.325. For the native *C. vermilara*, the qPCR runs resulted in *R* ^2^ = 0.998 with an efficiency of 96.3% and a slope of −3.414. The relative fluorescence unit threshold for all qPCR runs was set up at 300 RFU. Melt peaks under the threshold were not considered for further analysis. qPCR detection limits were estimated for each individual species, confirming detectability only if occurred within all three dilution triplicates, above 300 RFU, with 1 × 10^−4^ ng/µl for *C. fragile* and *C. tomentosum*, and 1 × 10^−6^ ng/µl for *C. vermilara*. Only the confirmed detection concentrations were used for standard curve calculations. All three positive controls amplified at species‐specific temperature melt peak at each qPCR run.

### 3.1 *C. tomentosum* ex situ experiment

**Figure 2:**
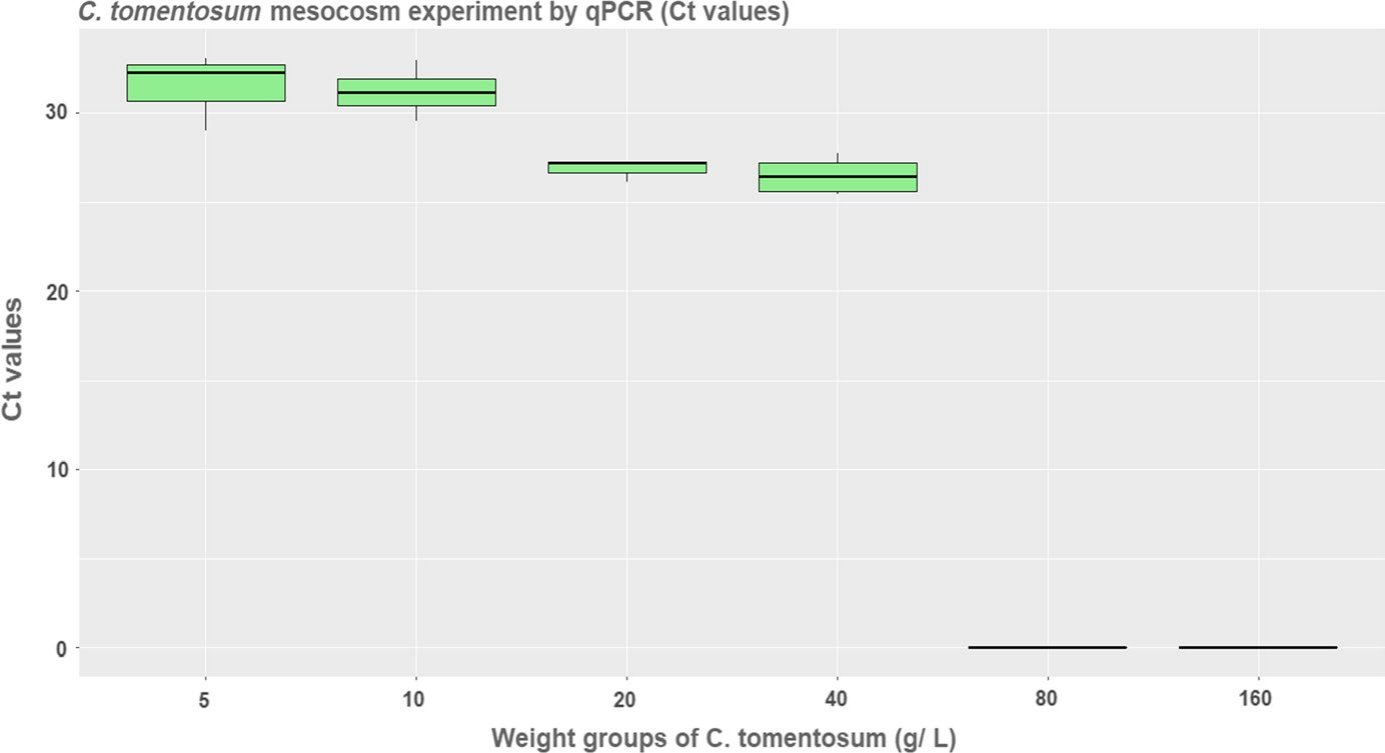
eDNA density (Ct values) correlated to *C. tomentosum* actual biomass (g/L) in the ex situ experiment collected from Cabo de Peñas sampling point

*C. tomentosum* eDNA density, based on Ct values (eDNA copies/µl), amplified until the biomass threshold of 80 g/l (Figure 2), which was the upper limit of detection by qPCR. Results of the Pearson correlation indicated that there was a significant negative association between the actual specimens’ biomass and the Cq values (*r*(19) = −0.884, *p* < 0.001), indicating a positive eDNA increase with the increase of specimen biomass, reaching a plateau between 20 and 40 g/L, with an average of 26.610 ± 0.861 Ct values (1.083 × 10^6^ ± 6.4 × 10^5^ eDNA copies/µl). The lowest and highest *C. tomentosum* eDNA densities measured in the field were 4.930 × 10^2^ up and 5.812 × 10^6^ eDNA copies/µl, which would correspond to an approximate density of 1.504 up to 47.66 g/L when compared to the ex situ experiment.

### 3.2 Spatial and seasonal variation

**Figure 3:**
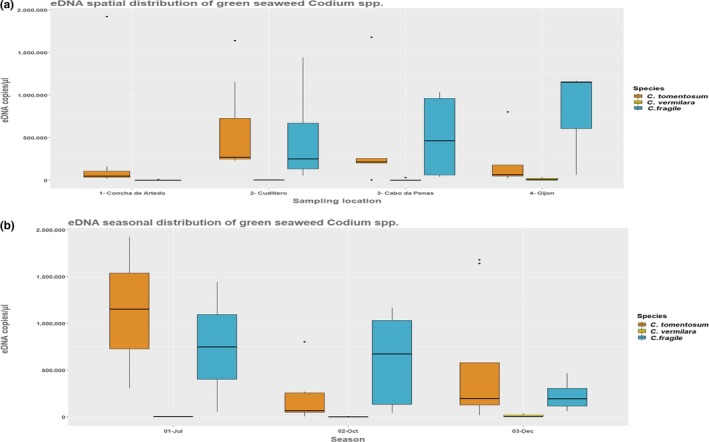
(a) Spatial and (b) seasonal density variation (eDNA copies/µl) of all three species, *C. fragile*, *C. tomentosum*, and *C. vermilara*. For spatial variation, samples from all sampling events conducted in July, September, and December were pooled. For seasonal variation, samples from all sampling stations were pooled

We evaluated the seasonal and spatial representation of *C. fragile*, *C. tomentosum*, and *C. vermilara* by qPCR quantification (Figure 3). Overall, the most predominant two species were *C. fragile* and *C. tomentosum*, the latter accounting for the highest abundance of eDNA copies of all the species, with an average of 6.079 × 10^5 ^eDNA copies/µl in the two Western sampling points and 2.201 × 10^5 ^eDNA copies/µl at the Eastern sampling side. *C. fragile* was predominantly found on the east with an average of 5.629 × 10^5 ^eDNA copies/µl and a more even distribution between the three localities with species occurrence (±6.653 × 10^4^ eDNA copies/µl), without spatially predominant patterns of *C. vermilara* eDNA presence (Figure 3). We did not find *C. fragile* at Concha de Artedo, the most Western sampling point, whereas the highest eDNA presence was found at both ports, Cudillero with an average of 32.956 ± 1.78 Ct values corresponding to 4.780 × 10^5^ ± 4.945 × 10^5^ eDNA copies/µl, and Gijon with 32.733 ± 2.348 Ct values, corresponding to 7.929 × 10^5^ ± 6.323 × 10^5^ eDNA copies/µl. We detected the highest average density of *C. fragile* in the summer, but the highest single eDNA detection was measured in October in the port of Gijon with 3.192 × 10^6^ eDNA copies/µl. The only locality where we found eDNA of *C. fragile* at all seasons was at the port of Cudillero, whereas in the port of Gijon we only detected it in the autumn sampling. *C. tomentosum* eDNA presence was detected at all four stations, with a highest coverage in the summer and winter periods (Figure 3). *C. tomentosum* exhibited the overall highest presence in summer and winter compared to other two species, whereas the abundance of *C. fragile* was high in summer and autumn and declined in winter (Figure 3). The highest abundance of *C. tomentosum* was detected in July at Concha de Artedo with 4.922 × 10^6^ ± 9.515 × 10^5^ copies/µl (24.814 ± 0.288 Ct value). eDNA from *C. vermilara* had been also found at all four stations with the highest representation in the winter, where on the average the eDNA copy number was 11.390% higher compared to autumn period (Figure 3). In the summer, we only detected at port of Cudillero with 32.023 ± 1.113 corresponding to 5.082 × 10^3^ ± 3.380 × 10^3 ^eDNA copies/µl.

Seasonal and spatial presence/absence of species assessment using binary logistic regression, testing for an interaction between species, sampling location, and season, indicated high variation between species (Table 2, χ^2^ = 87.978, *df* = 2, *p* < 0.001), location (Table 2, χ^2^ = 15.727, *df* = 3, *p* < 0.001), and sampling season (Table 2, χ^2^ = 24.752, *df* = 2, *p* < 0.001), with a significant interaction of species and location (Table 2, χ^2^ = 8.997, *df* = 5, *p* < 0.001). The second model, assessing presence/absence, testing for an interaction between species, artificial/natural location, and season, identified a higher overall presence of all species at the two artificial ports (Table 2, χ^2^ = 56.906, *df* = 1, *p* = 0.011). A density dependence linear model accounting for interactions between species, location, and season showed significant differences in density between species (Table 3, *F* = 12.468, *df* = 2, *p* < 0.001) due to *C. tomentosum* high and *C. vermilara* lower abundance (Tukey's post hoc test, *p* = 0.001) and sampling seasons (Table 3, *F* = 3.409, *df* = 2, *p* = 0.042), based on eDNA copies/µl. Significant density dependence interactions were identified among species and sampling season (Table 3, *F* = 3.617, *df* = 4, *p* = 0.013), in particular between low *C. vermilara* density in October and December compared to high *C. fragile* density in October and also *C. tomentosum* higher winter densities compared to *C. fragile* (Tukey's post hoc test, *p* < 0.011), and also between sampling season and location (*F* = 3.309, *df* = 4, *p* = 0.019), mainly due to low seasonal representation of species at Concha de Artedo compared to other localities at all sampled seasons (Tukey's post hoc test, *p* < 0.006). The second density dependence model assessed an interaction between artificial and natural segregation of specific species in seasons, and two significantly different relations were identified, the species‐specific density change within season and the artificial/natural segregation with seasonal changes (Table 3, *F* = 3.403, *df* = 4, *p* = 0.015; *F* = 3.939, *df* = 2, *p* = 0.025), respectively, with an average higher eDNA copies/µl found at the two artificial ports compared to the natural locations, particularly in autumn.

**Table 2:** Evaluation of seasonal and spatial patterns of all three species using binary logistic regression for species presence/absence assessment, identified with two models, first one based on species, sampling season, and location, and second one based on species, sampling season, and artificial/natural categories, including interactions between them

Factors of interactions	Deviance	*df*	χ^2^	*p*
Presence/absence = Species × Sampling season × Location				
Species	20.908	2	87.978	<0.001
Sampling season	24.752	2	63.225	<0.001
Location	47.798	3	15.727	<0.001
Species × Sampling season	0.078	4	15.727	0.9889
Sampling season × Location	0	4	6.730	1
Species × Location	8.997	5	6.730	<0.001
Species × Sampling season × Location	0	4	6.730	1
Presence/absence = Species × Sampling season × Artificial/natural				
Species	20.907	2	87.978	<0.001
Sampling season	24.752	2	63.225	<0.001
Artificial/natural	6.318	1	56.906	0.011
Species × Sampling season	8.001	4	48.903	0.091
Species × Artificial/natural	3.151	2	45.752	0.206
Sampling season × Artificial/natural	2.839	2	42.912	0.241
Species × Sampling season × Artificial/natural	0	4	42.912	1

Note

All sampling locations, Concha de Artedo, Cudillero, Cabo de Peñas, and Gijón, were included in the analysis.

**Table 3:** Evaluation of seasonal and spatial patterns of all three species using linear models based on Gaussian distribution for species abundance estimation by eDNA copies/µl

Factors of interactions	*F*	*df*	*p*
eDNA copies/µl = Species × Sampling season × Location			
Species	12.468	2	<0.001
Sampling season	3.409	2	0.042
Location	0.303	3	0.822
Species × Sampling season	3.617	4	0.013
Sampling season × Location	3.309	4	0.019
Species × Location	0.350	5	0.878
Species × Sampling season × Location	0.673	4	0.614
eDNA copies/µl = Species × Sampling season × Artificial/natural			
Species	12.088	2	<0.001
Artificial/natural	0.115	1	0.735
Sampling season	3.272	2	0.046
Species × Artificial/natural	0.103	2	0.902
Species × Sampling season	3.403	4	0.015
Sampling season × Artificial/natural	3.939	2	0.025
Species × Artificial/natural × Sampling season	0.045	2	0.955

Note

The first linear model (Species × Sampling season × Location) includes all three species, together with sampling season, location, and interaction terms between them, and the second model (Species × Artificial/natural × Sampling season) evaluates additional difference between the artificial/natural species‐specific seasonal distribution. All sampling locations, Concha de Artedo, Cudillero, Cabo de Peñas, and Gijón, were included in the analysis.

## 4 DISCUSSION

We used an eDNA approach to assess the spatio‐temporal variation of a non‐native algal species in relation to two of the closest native species, using eDNA absolute quantification approach in the Bay of Biscay at three different seasons and at four locations along an environmental longitudinal gradient, confirming previously defined distribution patterns of the two native, *C. vermilara* and *C. tomentosum*, including invasive *C. fragile* along the sampling sites (García et al., 2018; Skukan et al., 2017). The observed high eDNA density of *C. fragile* at both ports and its new detection at Cabo de Peñas confirms that this invasive species is spreading. The additional ex situ experiment of *C. tomentosum* contributed toward estimations of eDNA correlation with the relative density assessment in the field. eDNA density assessments using ex situ experiments have been used previously to estimate how relative abundance correlates with eDNA copies (Doi et al., 2015; Takahara, Minamoto, Yamanaka, Doi, & Kawabata, 2012; Wilcox et al., 2016), finding it as the most suitable measure for general biomass/density species‐specific assessment being reflected in eDNA relative densities. We found no *C. decorticatum* in our eDNA sampling, confirming previous studies along the coast (García et al., 2018), despite having been occasionally reported (Cires Rodríguez & Rico Ordás, 2007). Tide‐induced sampling limitations had been one of the potential causes proposed for finding no particular species during sampling events (Rojo et al., 2014), but our study suggests that this species was absent at the time of sampling as water sampling was not affected by the available shoreline sampling transect. *C. decorticatum* was not detected at all sampling events, as well as *C. fragile* was not detected at the most Western sampling point, which illustrates the usefulness of eDNA as a tool for seaweed monitoring. The east side higher density of *C. fragile* spread found with eDNA, overlapped with previous findings (Cires Rodríguez & Rico Ordás, 2007). Our results were also concordant with the previous surveillance at most western point of Concha de Artedo where in summer sampling events the majority of the specimens belonged to *C. tomentosum* with a small representation of *C. vermilara* and no confirmed presence of *C. fragile* (Rojo et al., 2014).

*C. fragile* are reproductively more successful in warmer waters with maximum growth at 24*°*C (Hanisak, 1979) compared to the two native ones with lower temperature preferences (Yang, Blunden, Huang, & Fletcher, 1997). This could explain the higher densities of *C. fragile* on the east side of Cantabrian coast due to higher summer temperatures modifying seaweed assemblages (Díez et al., 2012). Our results confirmed species‐specific seasonal and spatial overlap with previously defined distribution (García et al., 2018). *C. vermilara's* optimum growth occurs at 18 µmol/mol of photon irradiance (Yang et al., 1997) and averaged quarter and half of the averaged photon irradiance of other five *Codium spp*., making it an ideal candidate species to shift its reproductive cycle toward colder seasons. *C. fragile* becomes a dominant canopy‐forming species once established as dense meadows in new environments (Scheibling & Gagnon, 2006) and could force *C. vermilara* to shift toward winter growth preferences. Similar coexisting acclimatization of two native and invasive kelp species in the same environment has been previously evidenced, where habitat preferences were identified through specific gene expression in correlation with temperature shifts (Henkel & Hofmann, 2008). The results show that *C. fragile* was the predominant species during autumn sampling, whereas previously it had been predominantly found in the summer period (Rojo et al., 2014). Colder spring and summer temperatures in the year of the eDNA sampling, with additional warmer temperatures in autumn (only 1°C degree difference from summer sampling), could have postponed *C. fragile* reproductive season toward autumn, and the corresponding increase in release of gametes (Bohmann et al., 2014) might be correlated to the eDNA density increase in that particular autumn. With the increasing temperatures along the N Spanish coast (Gómez‐Gesteira, Decastro, Alvarez, & Gómez‐Gesteira, 2008), a range shift in the relative abundance of seaweed species (Duarte et al., 2013; Voerman, Llera, & Rico, 2013) and the potential increase of *C. fragile* toward the west could be expected. Years with increased coastal upwelling at the Central Cantabrian Coast could potentially increase the seaweed distribution, as observed for the planktonic phase of local barnacle populations (Rivera et al., 2013). High chlorophyll concentration levels in summer have also been observed to have a positive effect on settlement of another invasive seaweed, the Asian kelp *Undaria pinnatifida* along the Cantabrian coast (Báez et al., 2010). Both the upwelling and increased chlorophyll levels seem to be the result of prevailing northeast winds during summer (Botas, Fernández, Bode, & Anadón, 1990), which result in thermal stratification, that could have prolonged the seasonal persistence of *C. fragile*.

A high eDNA density of invasive *C. fragile* was detected in both ports, with potential displacement of the native species. Colonization of *C. fragile* subspecies on artificial marine structures is a regular occurrence around the globe (Bulleri & Airoldi, 2005; Campbell, 1999; Trowbridge, 1995), where artificial structures facilitate its spread. eDNA‐based methods could be used for invasive green seaweed monitoring, by integration with port baseline surveys (David, Gollasch, & Leppäkoski, 2013) for ballast water management or implementation within Marine Strategy Framework Directive (Borja, Elliott, Carstensen, Heiskanen, & Bund, 2010; Directive, 2008). Despite the apparent noncompetitive status of *C. fragile* in the Cantabrian Sea due to their clear seasonal reproductive segregation with native species (García et al., 2018), there is no potential reduction in its introduction rates, which depends on multiple vectors (Boudouresque & Verlaque, 2010) such as shipping routes through ports.

Substantially higher eDNA copy numbers were identified for *C. fragile* and *C. tomentosum* in comparison with *C. vermilara*, which had 100‐fold lower detection limits compared to the other two species. This could be explained by differences in qPCR efficiencies and variation in species‐specific detection limits (Ludwig & Schleifer, 2000), where for between species primer calibration identical sequences between the primer targets are required. It has also been shown that qPCR primers efficiencies do not vary between different species or strains (Matsuki, Watanabe, Fujimoto, Takada, & Tanaka, 2004), indicating high precision of the method used for the interspecific comparison of *Codium *spp. in the present study. PCR assays may not vary greatly, depending on the species or strains. By using copy numbers per µl of DNA, it should be possible to compare the results between species, research facilities (Whelan et al., 2003), provided the same chemistry is used (Dhanasekaran, Doherty, & Kenneth, 2010), instead of comparing Ct values, which lack species‐specific quantification precision. The ex situ experiment identified an upper limit of detection due to the selection of 1 ng/µl as the highest level of the standard curve dilution series in order to be able to detect low eDNA densities, representative for the values found in environment. Yet, the upper limit of detection was reached at concentrations that were unlikely to be found in the natural environment (Drouin, McKindsey, & Johnson, 2012; Scheibling & Gagnon, 2006). Primer specificity is important for successful detections of species (Mächler et al., 2014; Wilcox et al., 2013), but comparison of species densities using species‐specific primers should be interpret with caution, comparing eDNA efficiency with the traditional abundance estimates (Agersnap et al., 2017). Thus, as the ex situ experiment was only conducted on *C. tometosum*, it is possible that different upper or lower detection limits applied to the other two species, *C. fragile* and *C. vermilara*, resulting in different detection levels, as eDNA quantification can vary even for the same species under different conditions (Klymus, Richter, Chapman, & Paukert, 2015).An internal inhibition control to monitor for PCR inhibitors (Henke‐Gendo et al., 2012), was not added to each individual sample but could benefit toward qPCR run efficiency assessment.

Early detection of seaweed species in the aquatic environment can significantly improve AIS management and potential eradication (Jerde et al., 2013), with more efficient monitoring and containment of its spread (Tréguier et al., 2014), predicting its dispersal through spatial distribution models (Muha, Rodríguez‐Rey, Rolla, & Tricarico, 2017), or influencing management and policy decisions (Kelly et al., 2014). As we have demonstrated here, eDNA can be used to assess the spatial and seasonal distribution patterns of invasive and native green seaweed algae.

## 5 CONCLUSIONS

Our results on the distribution of native and invasive *Codium* species largely confirm those from more traditional surveillance methods, indicating that species‐specific eDNA qPCR is an efficient and effective tool for monitoring seaweed seasonal and spatial patterns. We found seasonal and spatial segregation in the presence of the invasive and two of the native *Codium *spp., potentially explaining the establishment success of the non‐native species.

## CONFLICT OF INTEREST

The authors declare that there is no conflict of interest regarding the publication of this article.

## AUTHOR CONTRIBUTIONS

TPM, RS, YJB, and SC conceived the study. SC and YJB supervised the research outcomes and provided guidance for the final outcome of the paper. JMR contributed to sampling activities and advised about the known Codium sp. local and seasonal patterns. TPM and RS conducted all the sampling activities. TPM conceived all the laboratory work and did the statistical analysis with the guidance of CGL. SC, CGL, EGV, YJB and JMR obtained the funding. TPM prepared the manuscript with the support of SC, to which all authors contributed suggestions and revisions. TPM and RS are currently Early Career Researchers. The work is a product of their PhD research activities.

## DATA ACCESSIBILITY

The data used for the following study are allocated in Table S1.
