# Supplementary material for: Contrasting seasonal and spatial distribution of native and invasive Codium seaweed revealed by targeting species‐specific eDNA
Source: Ecol Evol. 2019 Jul 10;9(15):8567–79. doi: 10.1002/ece3.5379 (PMC6686311; doi:10.1002/ece3.5379)
Supplement: Supplementary file 1 [file ECE3-9-8567-s001.docx]

**Supplementary material**


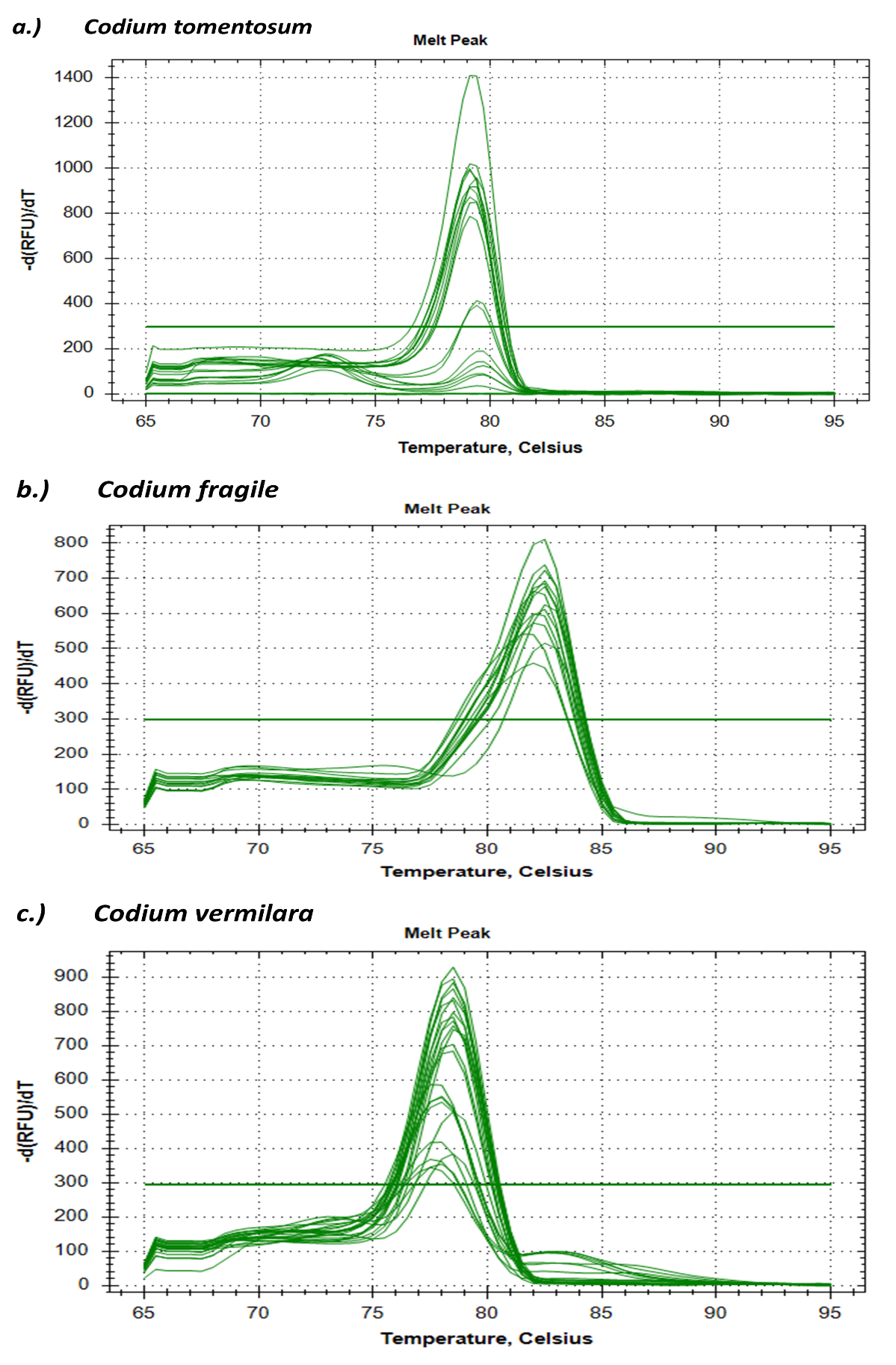


Fig S1.- qPCR melt peak temperatures for all three species a.) *C. tomentosum*, b.) *C. fragile, c.) C. vermilara*.

Table S1- Dataset used for seasonal and spatial distribution of *Codium spp.*

| Sampling season | Location | Species | Sampling point | eDNA technical average | Ct values average | Artificial/ natural |
| --- | --- | --- | --- | --- | --- | --- |
| 01-Jul | Concha de Artedo | C. tomentosum | CA1 | 2035895 | 24.76 | Natural |
| 01-Jul | Concha de Artedo | C. tomentosum | CA1 | 1922416 | 24.81 | Natural |
| 02-Oct | Concha de Artedo | C. tomentosum | CA2 | 39684.63 | 31.78 | Natural |
| 02-Oct | Concha de Artedo | C. tomentosum | CA2 | 49692.95 | 31.49 | Natural |
| 02-Oct | Concha de Artedo | C. tomentosum | CA2 | 53239.39 | 53239.40 | Natural |
| 03-Dec | Concha de Artedo | C. tomentosum | CA3 | 159446.1 | 30.29 | Natural |
| 03-Dec | Concha de Artedo | C. tomentosum | CA3 | 16379.3 | 34.25 | Natural |
| 03-Dec | Concha de Artedo | C. tomentosum | CA3 | 25073.77 | 32.42 | Natural |
| 01-Jul | Cudillero | C. tomentosum | CU1 | 1149953 | 26.91 | Artificial |
| 01-Jul | Cudillero | C. tomentosum | CU1 | 305656 | 30.05 | Artificial |
| 02-Oct | Cudillero | C. tomentosum | CU2 | 268938.1 | 29.07 | Artificial |
| 02-Oct | Cudillero | C. tomentosum | CU2 | 260128.4 | 29.04 | Artificial |
| 02-Oct | Cudillero | C. tomentosum | CU2 | 234794.5 | 29.31 | Artificial |
| 03-Dec | Cudillero | C. tomentosum | CU3 | 1640433 | 26.39 | Artificial |
| 03-Dec | Cudillero | C. tomentosum | CU3 | 226710.8 | 29.34 | Artificial |
| 02-Oct | Cabo da Penas | C. tomentosum | CP2 | 202690 | 29.41 | Natural |
| 02-Oct | Cabo da Penas | C. tomentosum | CP2 | 2930.44 | 36.10 | Natural |
| 02-Oct | Cabo da Penas | C. tomentosum | CP2 | 256268.3 | 28.94 | Natural |
| 03-Dec | Cabo da Penas | C. tomentosum | CP3 | 214412.3 | 29.32 | Natural |
| 03-Dec | Cabo da Penas | C. tomentosum | CP3 | 1679858 | 25.61 | Natural |
| 02-Oct | Gijon | C. tomentosum | G2 | 65908.17 | 31.06 | Artificial |
| 02-Oct | Gijon | C. tomentosum | G2 | 47147.13 | 31.64 | Artificial |
| 02-Oct | Gijon | C. tomentosum | G2 | 22336.43 | 32.63 | Artificial |
| 02-Oct | Gijon | C. tomentosum | G3 | 800615 | 28.09 | Artificial |
| 03-Dec | Gijon | C. tomentosum | G3 | 178942.2 | 29.31 | Artificial |
| 02-Oct | Concha de Artedo | C. vermilara | CA2 | 135.56 | 37.23 | Natural |
| 02-Oct | Concha de Artedo | C. vermilara | CA2 | 1567.83 | 34.69 | Natural |
| 02-Oct | Concha de Artedo | C. vermilara | CA2 | 110.34 | 37.55 | Natural |
| 03-Dec | Concha de Artedo | C. vermilara | CA3 | 388.11 | 35.88 | Natural |
| 03-Dec | Concha de Artedo | C. vermilara | CA3 | 6619.92 | 32.64 | Natural |
| 03-Dec | Concha de Artedo | C. vermilara | CA3 | 395.36 | 35.55 | Natural |
| 01-Jul | Cudillero | C. vermilara | CU1 | 2081.93 | 33.02 | Artificial |
| 01-Jul | Cudillero | C. vermilara | CU1 | 8753.14 | 31.14 | Artificial |
| 01-Jul | Cudillero | C. vermilara | CU1 | 4468.27 | 31.91 | Artificial |
| 02-Oct | Cudillero | C. vermilara | CU2 | 665.64 | 34.85 | Artificial |
| 02-Oct | Cudillero | C. vermilara | CU2 | 431.95 | 35.70 | Artificial |
| 02-Oct | Cudillero | C. vermilara | CU2 | 755.7 | 34.62 | Artificial |
| 03-Dec | Cudillero | C. vermilara | CU3 | 3828.51 | 32.05 | Artificial |
| 03-Dec | Cudillero | C. vermilara | CU3 | 4655.08 | 31.76 | Artificial |
| 03-Dec | Cudillero | C. vermilara | CU3 | 4685.09 | 31.74 | Artificial |
| 02-Oct | Cabo da Penas | C. vermilara | CP2 | 1198.86 | 33.85 | Natural |
| 02-Oct | Cabo da Penas | C. vermilara | CP2 | 75.82 | 38.44 | Natural |
| 02-Oct | Cabo da Penas | C. vermilara | CP2 | 368.87 | 37.96 | Natural |
| 03-Dec | Cabo da Penas | C. vermilara | CP3 | 31691.2 | 28.85 | Natural |
| 03-Dec | Cabo da Penas | C. vermilara | CP3 | 4747.479 | 31.72 | Natural |
| 03-Dec | Cabo da Penas | C. vermilara | CP3 | 4131.76 | 31.93 | Natural |
| 02-Oct | Gijon | C. vermilara | G2 | 328.7 | 35.88 | Artificial |
| 02-Oct | Gijon | C. vermilara | G2 | 36.7 | 36.70 | Artificial |
| 02-Oct | Gijon | C. vermilara | G2 | 350.7 | 35.78 | Artificial |
| 03-Dec | Gijon | C. vermilara | G3 | 25621.7 | 29.27 | Artificial |
| 03-Dec | Gijon | C. vermilara | G3 | 11811 | 31.30 | Artificial |
| 03-Dec | Gijon | C. vermilara | G3 | 36433.7 | 28.64 | Artificial |
| 01-Jul | Cudillero | C.fragile | CU1 | 1439625 | 30.70 | Artificial |
| 01-Jul | Cudillero | C.fragile | CU1 | 53199.11 | 34.99 | Artificial |
| 02-Oct | Cudillero | C.fragile | CU2 | 672077.7 | 35.11 | Artificial |
| 02-Oct | Cudillero | C.fragile | CU2 | 133067.9 | 32.05 | Artificial |
| 02-Oct | Cudillero | C.fragile | CU2 | 665175.9 | 31.34 | Artificial |
| 03-Dec | Cudillero | C.fragile | CU3 | 133553.8 | 33.80 | Artificial |
| 03-Dec | Cudillero | C.fragile | CU3 | 250007 | 32.70 | Artificial |
| 02-Oct | Cabo da Penas | C.fragile | CP2 | 962706.9 | 30.80 | Natural |
| 02-Oct | Cabo da Penas | C.fragile | CP2 | 1029995 | 31.10 | Natural |
| 02-Oct | Cabo da Penas | C.fragile | CP2 | 40338.99 | 35.39 | Natural |
| 03-Dec | Cabo da Penas | C.fragile | CP3 | 60549.1 | 34.80 | Natural |
| 03-Dec | Cabo da Penas | C.fragile | CP3 | 131067.9 | 32.03 | Natural |
| 03-Dec | Cabo da Penas | C.fragile | CP3 | 464526.5 | 31.84 | Natural |
| 02-Oct | Gijon | C.fragile | G2 | 1166408 | 32.20 | Artificial |
| 02-Oct | Gijon | C.fragile | G2 | 62206.77 | 35.30 | Artificial |
| 02-Oct | Gijon | C.fragile | G2 | 1150190 | 30.70 | Artificial |
